# Unilateral biportal endoscopy (UBE) spine surgery for thoracolumbar intervertebral disc diseases in dogs: An ex vivo cadaveric and in vivo preclinical study

**DOI:** 10.1111/vsu.14324

**Published:** 2025-08-04

**Authors:** Sanghyun Nam, Youngjin Jeon, Jueun Kim, Jaemin Jeong, Seongmok Jeong, Youngwon Lee, Haebeom Lee

**Affiliations:** ^1^ College of Veterinary Medicine Chungnam National University Daejeon Republic of Korea; ^2^ Department of Orthopedic Surgery Baroseomyeon Hospital Busan Republic of Korea

## Abstract

**Objective:**

To determine optimal portal position for thoracolumbar unilateral biportal endoscopy (UBE) spine surgery in dogs.

**Study design:**

Experimental ex vivo cadaveric and in vivo preclinical study.

**Animals:**

Seven cadavers and three healthy purpose‐bred Beagles.

**Methods:**

In the ex vivo phase, thoracolumbar mini‐hemilaminectomy was performed at T13–L1, L1–L2, and L2–L3 in seven cadavers using two UBE portal positions. The distance (X) from the spinous process to the mammillary process was measured fluoroscopically. Group A portals were placed from X to 2X; Group B portals from 2X to 3X. Visualization and instrument accessibility were compared. Based on cadaveric results, mini‐hemilaminectomy was performed in three live dogs using the superior (Group B) portal position. Intraoperative epidural pressure was measured. Postoperative magnetic resonance image (MRI) on day 0, 14, and 28 evaluated muscle recovery, fluid extravasation, and spinal/dural compression.

**Results:**

Mini‐hemilaminectomy was successfully performed with both portal positions. Surgical time and number of fluoroscopic scans did not differ between groups. Scope insertion angles were steeper in Group B (*p* < .001), which also showed higher visualization and accessibility scores (*p* < .001). Group B portals were used in the in vivo study. Epidural pressure remained stable, and MRI revealed transient postoperative muscle edema that resolved by day 28.

**Conclusion:**

UBE was feasible in cadavers and safe in live dog models. Group B portal positioning provided better visualization and accessibility.

**Clinical significance:**

UBE presents a potential minimally invasive approach for thoracolumbar spinal surgery in dogs.

**Abbreviations**
CT: computed tomography
IVDD: Intervertebral disc disease
MRI: magnetic resonance imaging
MP: mammillary process
MED: microendoscopic discectomy
MHL: mini‐hemilaminectomy
MISS: minimally invasive spine surgery
TL: thoracolumbar
UBE: unilateral biportal endoscopic

## 1 INTRODUCTION

Intervertebral disc disease (IVDD) is one of the most common neurologic diseases in dogs.^1^ Within the thoracolumbar (TL) region, T12–L3 segment is the most commonly affected.^1^, ^2^, ^3^, ^4^ Dogs with IVDD may be managed either medically or surgically, depending on severity and type of the condition.^5^, ^6^ Conservative therapy, including activity restriction, analgesia, and anti‐inflammatory medications, may be appropriate for dogs with mild deficits and disc protrusion.^5^, ^6^ Conversely, surgical decompression is generally recommended for dogs with disc extrusion and non‐ambulatory paraparesis or worse. Hemilaminectomy or mini‐hemilaminectomy is commonly performed to remove extruded disc material and relieve spinal cord compression.^6^

Mini‐hemilaminectomy (MHL) is a surgical procedure in which the accessory process is removed and the pedicle is partially resected cranially and caudally to create a small bony window, while preserving the facet joint. This procedure allows access to the spinal canal and facilitates decompression of the spinal cord and nerve roots.^7^ While MHL allows direct access to the affected disc and spinal canal, the open approach requires extensive muscle dissection, which increases iatrogenic tissue trauma.^8^, ^9^ To address these limitations, various minimally invasive spine surgery (MISS) techniques have been studied in veterinary medicine, including microscope surgery,^9^ endoscope‐assisted surgeries,^10^ uniportal endoscopy,^11^, ^12^, ^13^ microendoscopic discectomy (MED),^14^, ^15^ and the use of three‐dimensional (3D)‐printed guides for endoscope assisted spine surgery.^16^

Each MISS approach has strengths and drawbacks. Uniportal endoscopy, which uses a single portal for visualization and instrumentation, restricts instrument maneuverability and requires specialized equipment.^13^ MED and endoscope‐assisted techniques enhance visualization but still involve an open surgical approach and often experience interference between the endoscope and instruments, prolonging the surgical time.^14^

Over the past decades, MISS has advanced significantly in human medicine, including full endoscopic surgery and microscopic surgery, due to its benefits in reducing soft tissue disruption, postoperative pain, and recovery time.^17^, ^18^, ^19^, ^20^, ^21^, ^22^, ^23^ More recently, unilateral biportal endoscopic (UBE) spine surgery has emerged as a novel technique that utilizes two independent portals and a triangulation method similar to arthroscopy.^24^, ^25^ One portal accommodates the endoscope, and the other accommodates surgical instruments, allowing independent visualization and instrument maneuverability.^24^ A crucial aspect of UBE is the optimal portal placement, as it directly influences visualization, instrument angle and reach, and overall procedural efficiency.^24^, ^26^ Despite its increasing use in human medicine, the application of UBE in veterinary medicine remains largely unexplored.

This study aimed to identify the optimal portal position for TL MHL using UBE in cadaveric models and to preclinically evaluate the safety of UBE in an in vivo study. The in vivo assessment focused on evaluating changes in epidural pressure during surgery caused by continuous irrigation, the effectiveness of intraoperative bleeding control, and potential postoperative spinal cord and muscle injury postoperatively. The first hypothesis was that MHL using UBE is feasible in dogs, with no difference in visualization and performance between portal positions. The second hypothesis was that the UBE is safe in live dogs, without affecting epidural pressure or postoperative neurologic function.

## 2 MATERIALS AND METHODS

### 2.1 Ex vivo study

#### 2.1.1 Cadaver specimens and preparation

Prior to the main cadaveric study, three client‐owned mongrel dogs that had been euthanized for clinical reasons unrelated to this study were donated to our institution after obtaining consent from their owners. These three mongrel cadavers were used exclusively in a pilot study aimed at refining the surgical technique and establishing the experimental protocol and were not included in the main experiment analysis.

The seven male Beagle cadavers with mean bodyweight of 10.74 ± 0.48 kg were included in the main study. These cadavers were obtained following unrelated terminal studies conducted under approved protocols by the Institution Animal Care and Use Committee of Chungnam National University (approval nos.: 202304A‐CNU‐011 and 202404A‐CNU‐066). Prior to inclusion, radiographic and computed tomography (CT) images of the TL spine (T5–L5) were obtained to confirm vertebral integrity and the absence of spinal disease. Specimens were stored at – 20°C and thawed at room temperature for 24 h prior to surgery.

#### 2.1.2 Experimental design

**FIGURE 1:**
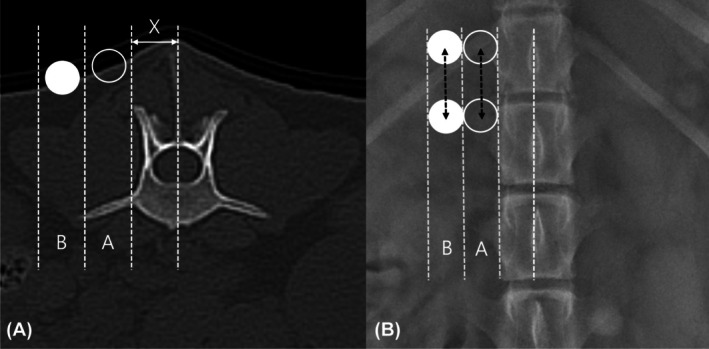
Portal positioning for mini‐hemilaminectomy (MHL) in the ex vivo study. (A) Transverse computer tomography (CT) image showing the measurement of portal distance using X, defined as the distance from the spinous process to the mammillary process (MP). Based on this measurement, Group A portal (empty circle) was positioned laterally at a distance of X from MP, and Group B portal (filled circle) were placed an additional distance of X further laterally. (B) Dorsoventral radiographic image illustrating the same portal positions, with two portals each for Group A (empty circle) and Group B (filled circle). The instrument and endoscope portals were spaced 2 cm apart to enable triangulation (dotted double arrow).

Two portal positioning strategies were evaluated for their effectiveness in performing MHL at T13–L1, L1–L2, and L2–L3 levels. Portal positions were defined by adapting human anatomical landmarks to canine anatomy.^24^, ^26^ Locations were determined fluoroscopically by measuring distance X (Figure 1A) from the spinous process to the mammillary process (MP) in a dorsoventral position.

**FIGURE 2:**
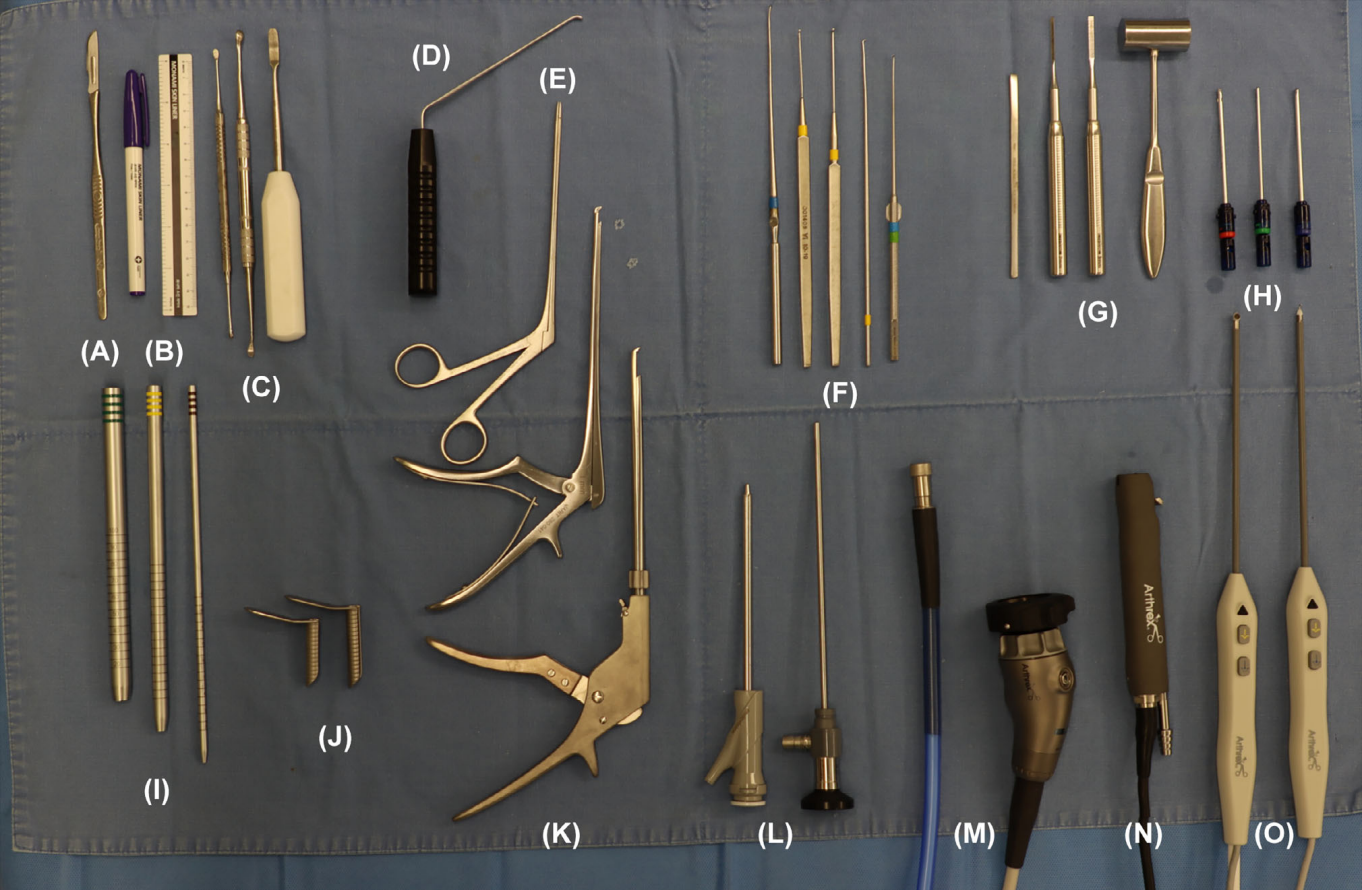
Surgical instruments for performing biportal endoscopic spine surgery. (A) Scalpel and blade for skin incision. (B) Surgical pen and ruler for portal planning. (C) Periosteal elevator for muscle elevation. (D) Root retractor (Endovision) for nerve root retraction. (E) Pituitary forceps (Solendos) for disc material removal. (F) Ball tip probe for palpation of anatomical landmark. (G) Chisel and mallet for initial bone entry. (H) A 3 mm hooded bur and shaver system (Arthrex) for bone removal (I) Serial portal dilator (Solendos) for creating the working channel. (J) Semi‐tubular retractor (Solendos) to maintain the outflow. (K) A 1 mm Kerrison rongeur (Integra Jarit GmbH) and rotation Kerrison rongeur. (Solendos). (L) 0° 4.0 mm scope (Endovision). (M) Camera and light cable (Synergy, Arthrex). (N) Saber shaver (Arthrex). (O) Bipolar radiofrequency (RF) probe system for soft tissue removal and coagulation (Apollo RF probe, Arthrex).

In Group A, both portals were placed laterally at a distance of X from the MP. In Group B, they were positioned farther laterally, starting from the lateral end of Group A and extending out to 2X from MP. In both groups, incisions were 1 cm in size and spaced 2 cm apart (Figure 1B), positioned slightly cranial or caudal relative to each other to facilitate triangulation and prevent instrument collision. The surgical table setup is shown in Figure 2.

In each cadaver, the left and right sides at each disc level were randomly assigned to either Group A or B, ensuring a balanced distribution of portal positions across different levels and sides. A total of 42 MHL procedures were performed in seven cadavers, with equal and random allocation of portal positions.

#### 2.1.3 Ex vivo experiment

**FIGURE 3:**
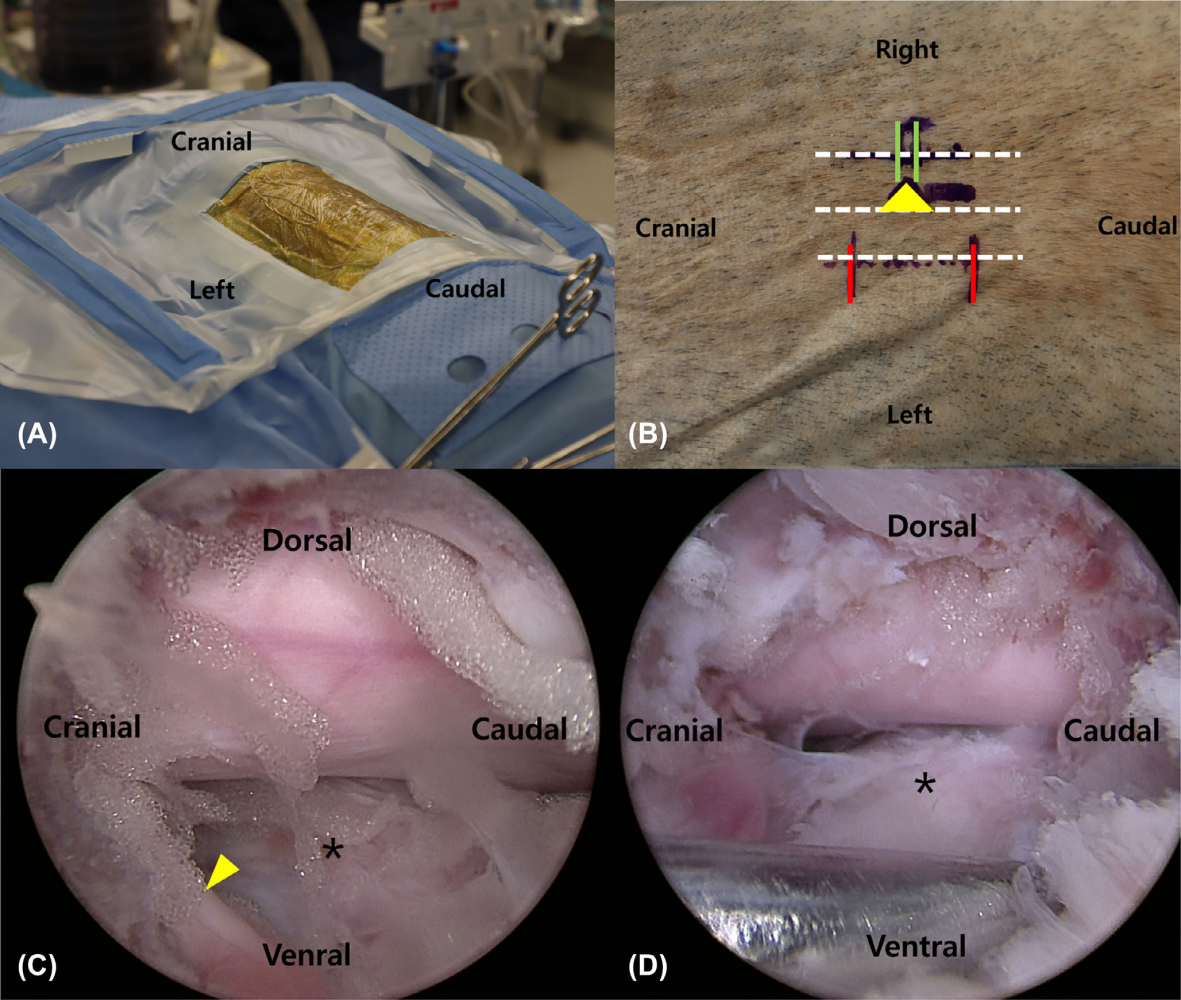
Ex vivo model preparation and improved intraoperative visualization. (A) Cadaver positioned in sternal recumbency with the surgical field prepared. (B) Close‐up of the cadaver's left thoracolumbar region with skin markers: Red line indicates the planned trajectory of the instrument portal, the green line marks the intervertebral disc space level, and the yellow arrowhead points to the location of the intervertebral foramen. (C) Endoscopic visualization of the surgical field after mini‐hemilaminectomy via Group B portals: The nerve root (arrowhead) is visualized, and intervertebral disc space (asterisk) is clearly visible beneath spinal cord. (D) Ventral view of endoscopic visualization. Nerve root (arrowhead) is retracted gently with a probe, providing an open view of the intervertebral disc space (asterisk).

Cadavers were positioned in sternal recumbency on a vacuum beanbag. The surgical site was prepared using an endoscopic drape (Endoscopy drape UBE, Sejong Healthcare, Gyeonggi‐do, Republic of Korea) (Figure 3A). All procedures were performed by a single right‐handed surgeon (SN).

Anatomical landmarks, including the lateral border of the MP, spinous process, and intervertebral disc space were marked fluoroscopically (Figure 3B). Portal boundaries for Groups A and B were confirmed as previously described (Figure 1).

After landmark identification, two 1 cm skin and fascial incisions were made 2 cm apart using a No. 10 scalpel blade. On the left side, the cranial portal was used for viewing and the caudal portal for instrumentation. On the right side, the arrangement was reversed. A serial dilation sleeve (Solendos Inc., Seoul, Republic of Korea) was inserted through the instrument portal to the level of the pedicle. The sheath of a 0° 4.0 mm endoscope connected with obturator (Endovision, Daegu, Republic of Korea) was inserted through the viewing portal. Once the placement was verified, the epaxial muscle was bluntly elevated from the pedicle.

After muscle elevation, the obturator was removed and a camera with light source (Arthrex, Naples, Florida) was inserted. A 40 mm semi‐tubular retractor (Solendos) was placed over the dilation sleeve. The position was confirmed using the fluoroscopy. Residual soft tissue was cleared using the RF probe. Fluoroscopic scans were taken as needed to confirm anatomical orientation during portal placement and scope/instrument insertion, particularly when spatial orientation was uncertain or temporarily lost.

**FIGURE 4:**
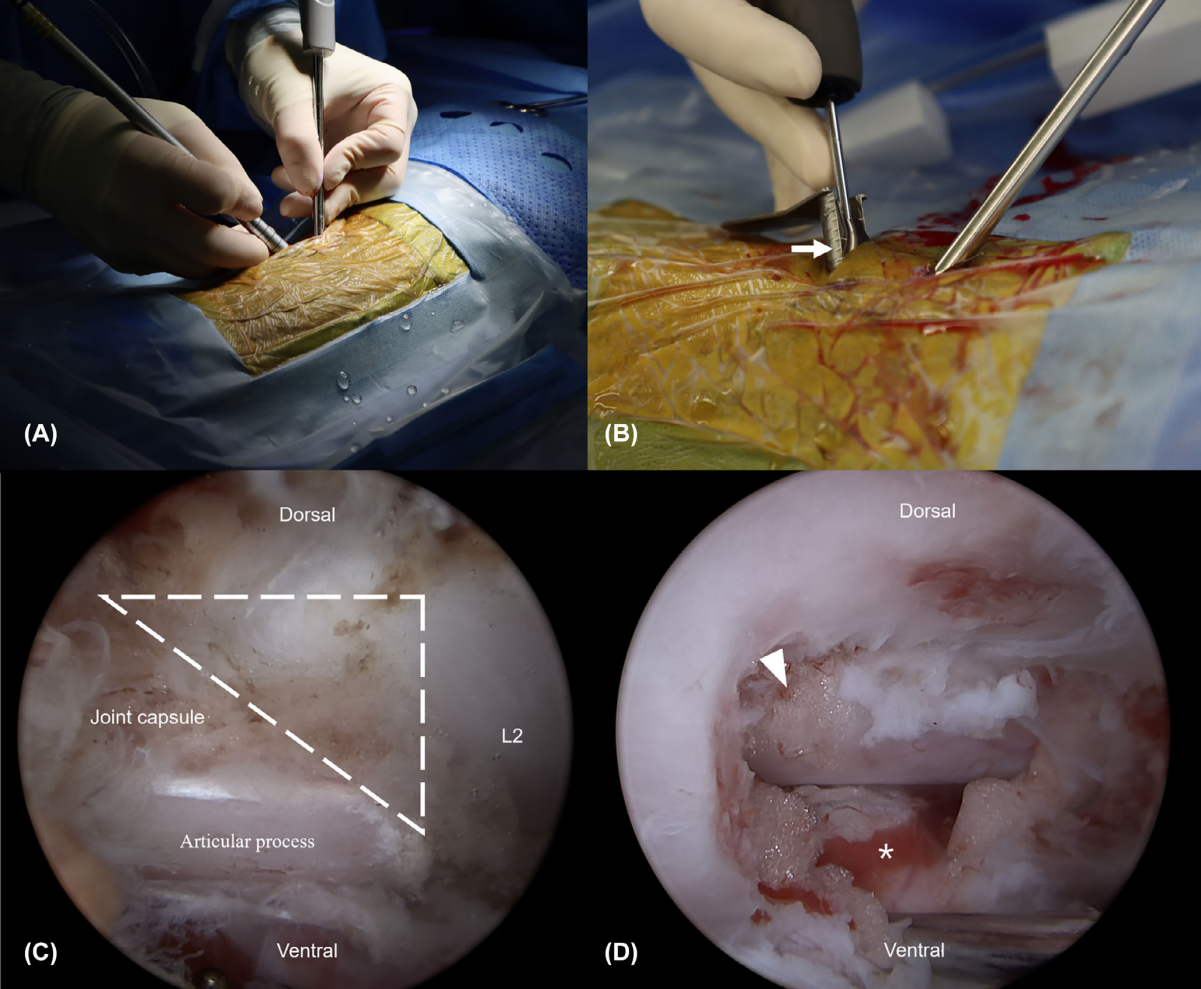
In vivo intraoperative description. (A) Triangulation technique. (B) Outflow (arrow) must be maintained during the procedure for visualization and surgical safety. Placement of semi‐tubular retractor may help maintain the outflow. (C) Identification of anatomical landmark. The accessory process is visualized as an anatomical landmark. The dashed triangle represents the modified Kambin's triangle, working corridor for accessing the intervertebral disc space. (D) Intraoperative view after mini hemilaminectomy. The venous sinus (asterisk) is visible on the ventral side of the spinal cord. The epidural fat (arrowhead).

To maintain a clear surgical view, 0.9% normal saline was connected to the scope sheath for continuous flow. Outflow (Figure 4B) was confirmed through the instrument portal. To maintain adequate irrigation pressure, a 1 L fluid bag was positioned 40 cm above the surgical table, generating an irrigation pressure of 29.4 mmHg. This pressure was calculated using the hydrostatic pressure formula:

$$\begin{array}{l} \text{Pressure}\;\left(\text{Pascal}\right)=\text{height}\;\left(m\right)\times \text{density}\;\left(\mathrm{kg}/m^{3}\right)\\ \;\times \;\text{gravity}\;\left(m/s^{2}\right) \end{array}$$

where the density of water is 1000 kg/m^3^, gravitational acceleration is 9.81 m/s^2^, and 1 mmHg = 133.322 Pascal.

After establishing the working zone and identifying the accessory process as a landmark, MHL was performed using an arthroscopic hooded bur (Arthrex). The intervertebral disc was then probed to evaluate the accessibility.

#### 2.1.4 Ex vivo intraoperative scoring and time assessment

Visualization and accessibility of key landmarks were scored on a scale from 0 to 3 (Table 1). Key landmarks included the ventral border of the caudal articular process, foramen, nerve root, intervertebral disc space, and shoulder and axilla region of the nerve root (Figure 3). Intraoperative visualization was independently evaluated by three observers, including the primary surgeon (SN) and two additional observers (YJ, HB), using recorded endoscopic video. To ensure consistency, the primary surgeon instructed each observer individually.

**TABLE 1:** Ex vivo intraoperative visualization and accessibility score.

Score	Visualization	Accessibility
0	Cannot identify	Cannot access with probe
1	Partially visible	Significantly limited access with probe
2	Visible with effort	Moderate difficulty in access with probe
3	Clearly visible	Excellent access with probe

In contrast, accessibility was assessed intraoperatively by a single evaluator (SN), who performed all procedures. Real‐time assessment was necessary to evaluate dynamic aspects of the surgical field, including portal depth, anatomical access, and instrument maneuverability, which cannot be assessed through still images or recorded videos.

Procedure time was recorded from the initial anatomical landmark identification to closure. Phase 1 involved fluoroscopic identification of foramen; Phase 2 focused on working zone creation at Kambin's triangle (an anatomical corridor commonly referenced in human spine surgery),^27^, ^28^ a landmark commonly used in human medicine; and Phase 3 evaluated bone work.

#### 2.1.5 Statistical analysis

Statistical analysis was performed to compare visualization scores, accessibility scores, total surgical time, and the scope insertion angle between Groups A and B. The Wilcoxon signed‐rank test was used to compare total surgical time, scope angle, number of fluoroscopic scans, and accessibility scores between groups. For post hoc comparisons between groups, the Mann–Whitney U test was used as a non‐parametric test suitable for two independent samples. Interobserver agreement on visualization was assessed using Fleiss's κ. As the data in Group A did not follow normal distribution, the average scores for each specimen between groups were analyzed using the Wilcoxon signed‐rank test. The Wilcoxon signed‐rank test was conducted to evaluate whether the surgeon's dominant hand influenced procedure duration. A *p*‐value of .05 was the threshold for statistical significance. All statistical analyses were conducted using SPSS software (IBM SPSS Statistics, version 29, IBM Corp., Chicago, Illinois).

### 2.2 In vivo study

#### 2.2.1 Animals and preparation

The study was approved by the Institutional Animal Care and Use Committee of Chungnam National University (approval no.: 202501A‐CNU‐024). Three healthy purpose‐bred adult Beagle dogs were included in the in vivo study. The dogs were individually housed in kennels (1.5 × 2 × 2.5 m) and fed a commercially available maintenance diet. All dogs underwent thorough physical and neurologic examinations prior to the procedure. The inclusion criteria required that all animals be clinically healthy and exhibit no evidence of orthopedic or neurologic abnormalities. All three dogs met these criteria and were included in the study.

For pre‐ and postoperative magnetic resonance imaging (MRI) (Vantage Elan 1.5 T, Canon medical system Korea, Seoul, Republic of Korea), all animals received midazolam (0.2 mg/kg IV) as premedication. General anesthesia was induced with alfaxalone (1.5–4.5 mg/kg IV) and maintained with isoflurane in 100% oxygen. MRI images were obtained from C1 to C4 and T11 to L3 levels in each dog, including sagittal and transverse planes, using T1‐weighted, T2‐weighted, fat saturation (FatSat), and fluid‐attenuated inversion recovery (FLAIR) sequences. To ensure consistency, all imaging procedures were conducted by a single radiologist (YL).

#### 2.2.2 In vivo experiment design

The optimal portal position identified from the ex vivo models (Group B) was used for all in vivo procedures. Each dogs underwent surgery at one intervertebral level on the left side. Distance X, as defined in the ex vivo study, was premeasured in the preoperative MRI to ensure precise portal position during surgery.

All animals received midazolam (0.2 mg/kg IV) as premedication and general anesthesia for surgery was induced with alfaxalone (1.5–4.5 mg/kg IV) and maintained with isoflurane in 100% oxygen. Remifentanil (5 μg/kg/h IV) was administered during the surgery for analgesia. The surgical technique and patient positioning for the in vivo models were the same as those described in the ex vivo models, and standard aseptic preparation of the surgical site was performed.

#### 2.2.3 In vivo experiment

Cervical epidural pressure was selected for measurement due to its anatomical accessibility and previous reports in human medicine suggesting that increased pressure in the lumbar thecal sac during continuous irrigation may be transmitted cranially, potentially elevating cervical and intracranial pressures and resulting in complications such as neck pain, headache, or seizure.^29^, ^30^, ^31^, ^32^

Dorsal approach to the C1–2 vertebral foramen was performed in each dog by a single surgeon (YJ) to allow placement of an epidural pressure monitoring catheter. The C1–C2 foramen was exposed, and a Tuohy needle was inserted through the foramen. A flexible cervical catheter (GH Bio, Gyeonggi‐do, Republic of Korea) was then advanced caudally through the needle into the epidural space and connected to a pressure transducer (ACE medical, Seoul, Republic of Korea) to enable real‐time monitoring of epidural pressure throughout the procedure.

The MHL was then performed by the surgeon (SN) following the same protocol as described in previous ex vivo study and intraoperative endoscope video is available at supplemental material (Video Clip S1).

Postoperative pain management included CRI of remifentanil (5 μg/kg/h IV) for one day along with meloxicam (0.1 mg/kg subcutaneously once a day) and gabapentin (10 mg/kg orally twice a day) for seven days. Dogs were monitored daily for 28 days postoperatively to assess pain, neurologic status, and wound healing. Cage rest was maintained for 14 days. Follow‐up assessments of pain, gait, and neurologic function were performed up to one month postoperatively.

#### 2.2.4 In vivo postoperative imaging studies

Postoperative thoracolumbar region (T11–L3) MRI was performed on postoperative day 0, 14 and 28 to assess epaxial muscle recovery, fluid accumulation in the epaxial muscle, and potential cord injury or compression. The imaging protocol included sagittal and transverse planes using T1–weighted, T2‐weighted, fat saturation (FatSat), and FLAIR sequences.

On postoperative day 0, additional cervical 1–2 level MRI was performed to assess potential complications related to dural sac compression due to continuous irrigation.^30^ The imaging protocol included sagittal and transverse planes using T1‐weighted, T2‐weighted, fat saturation (FatSat), and FLAIR sequences.

## 3 RESULTS

### 3.1 Ex vivo models

Seven cadaveric specimens (*n* = 7) were included in the study. All were male Beagles with a mean bodyweight of 10.74 ± 0.48 kg.

### 3.2 Ex vivo scoring and time assessment

**FIGURE 5:**
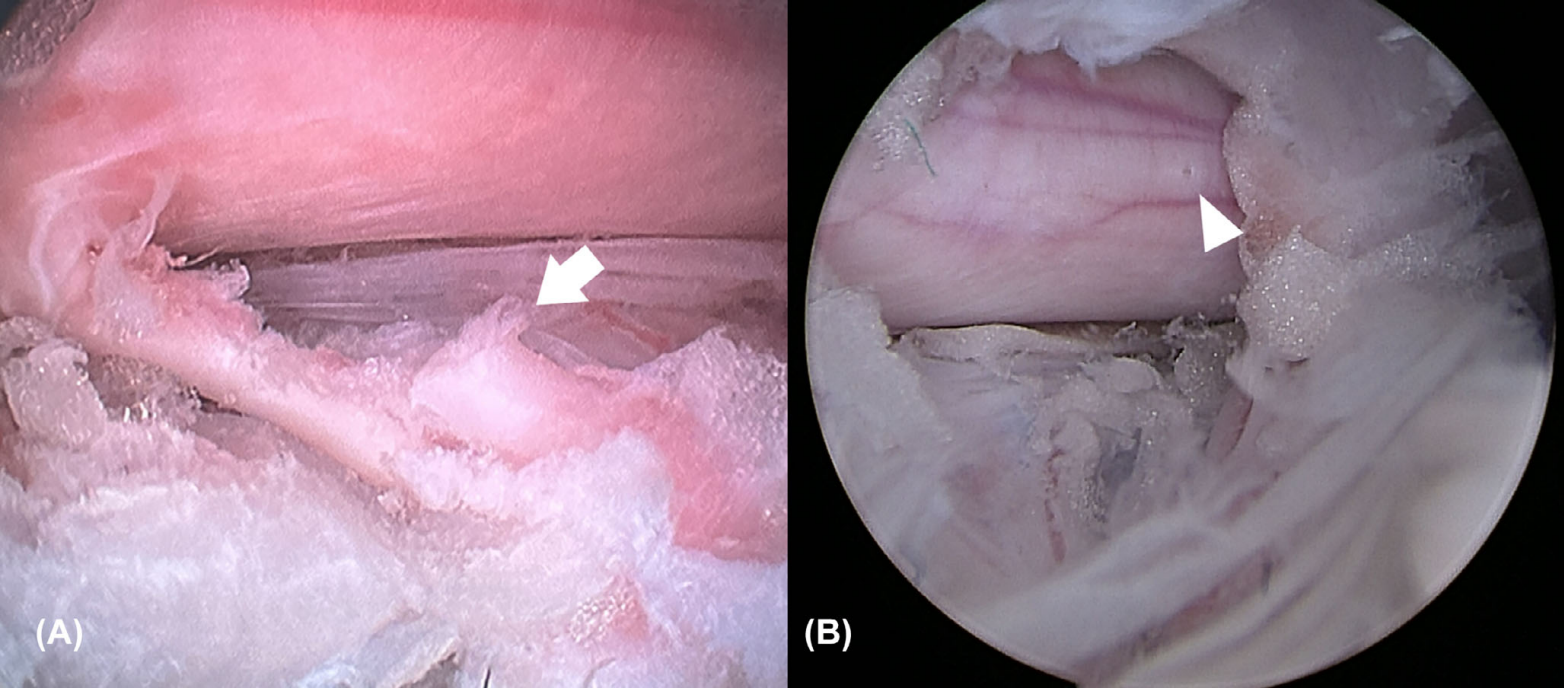
Ex vivo intraoperative complications. (A) Partial transection of nerve root in Group A. (B) Dura tear observed intraoperatively in Group A.

Group A and B did not differ in total surgical time (*p =* .602) or the number of fluoroscopic scans (*p* = .299). However, Group B exhibited a higher endoscope insertion angle compared to Group A (*p* < .001). Accessibility scores were higher in Group B (*p <* .001). Interobserver agreement for visualization scores was assessed according to the strength of agreement criteria proposed by Landis and Koch.^33^ Based on this classification, the agreement was slight in Group A (κ = 0.11) and fair in Group B (κ = 0.208) and visualization scores were higher in Group B (*p* < .001). Two nerve root injuries and one dura tear were observed in two specimens from Group A (Figure 5), whereas no complications were noted in Group B. The mean surgical time for the right and left side did not show significant difference (*p =* .695). Descriptive data of Group A and B are summarized in Table 2.

**TABLE 2:** Comparison of visualization score, accessibility score, surgical time, scope angle, fluoroscope scan number, complication between Group A and Group B in the ex vivo study.

Group	Visualization	Accessibility	Surgical time (min)	Scope angle (°)	Fluoroscope Scan	Complication
Group A	11.36 ± 2.56 ^a^	10.82 ± 2.63 ^b^	42.41 ± 20.06	42.55 ± 12.06 ^c^	1.95 ± 1.17	Dura tear (*n* = 1) Nerve root injury (*n* = 1)
Group B	13.05 ± 2.68 ^a^	12.91 ± 2.74 ^b^	41.45 ± 19.41	51.77 ± 11.87 ^c^	1.68 ± 0.72	‐

*Note*: ^a,b,c^ *p‐*value of Wilcoxon signed‐rank test statistically showing significant differences between groups. *p* < .05 was set as significant differences.

### 3.3 In vivo models

Three dogs were included in the in vivo study, consisting of two females and one male. The mean bodyweight of dogs was 9.77 ± 0.21 kg. All dogs maintained normal neurologic function and gait throughout the one month postoperative period, with no abnormalities detected during follow‐up assessments. Postoperative fluid extravasation resolved by postoperative day 14.

### 3.4 In vivo surgical procedures

The mean surgical time was 78.67 ± 22.6 min. All procedures were performed on the left side, and no intraoperative complications were observed. Physiological parameters, including heart rate, respiratory rate, blood pressure, and oxygen saturation, and end tidal CO_2_ remained within normal limits throughout the procedures, indicating stable anesthesia. The mean cervical epidural pressure during UBE procedure was 2.0 ± 1.0 mmHg, which is within the previous reported normal baseline range (2.1 ± 6.1 mmHg).^34^

### 3.5 Imaging studies

**FIGURE 6:**
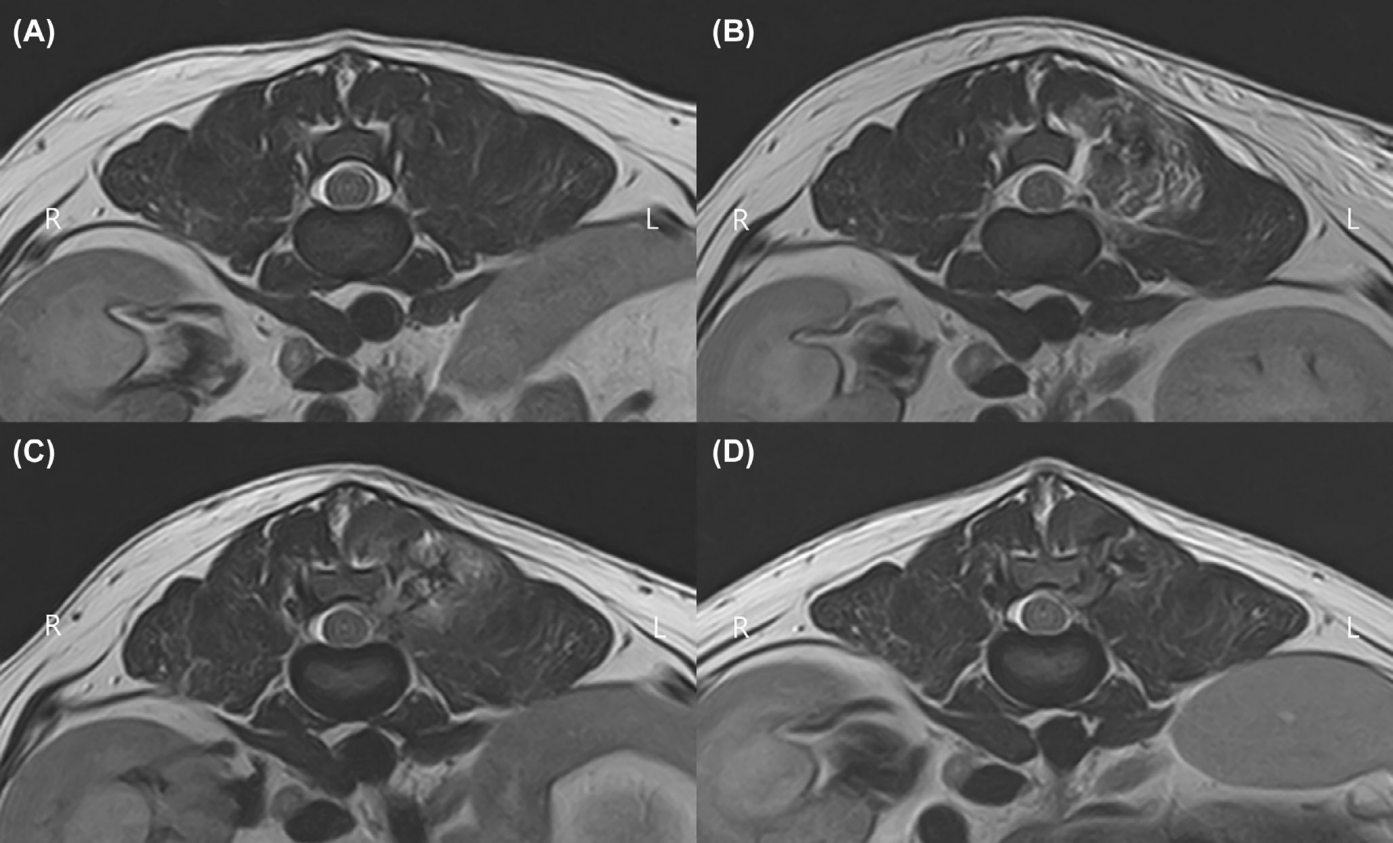
Serial transverse T2‐weighted magnetic resonance image (MRI). (A) Preoperative MRI. Normal anatomical structures with no abnormal signal intensity observed in the thoracolumbar region. (B) Postoperative day 0. A localized hyperintense signal is visible in the left epaxial muscle surrounding the surgical site, suggesting postoperative edema and fluid accumulation. No significant signal changes are noted in the spinal cord. (C) Postoperative day 14. The hyperintense signal in the epaxial muscles has decreased in both intensity and extent compared to postoperative day 0, indicating partial resolution of edema and inflammation. (D) postoperative day 28. Further reduction in signal intensity and extent is observed, with near‐complete resolution of fluid‐related changes and muscle damages.

Preoperative transverse T2‐weighted MRI (Figure 6A) showed normal anatomical structures with no abnormal signal intensity. On postoperative day 0, T2‐weighted MRI (Figure 6B) revealed localized hyperintense signal in the epaxial muscles around the surgical site, consistent with postoperative edema and fluid accumulation. No spinal cord signal change indicating neural injury was observed. FLAIR sequences showed subtle fluid retention between the bone and epaxial muscle.

By postoperative day 14, the hyperintense signal in the epaxial muscles had decreased (Figure 6C), suggesting a resolution of acute inflammation, although residual signal remained, indicating ongoing edema. Notably, fluid retention on FLAIR sequences had resolved by postoperative day 14.

By postoperative day 28, T2‐weighted image (Figure 6D) demonstrated near‐complete resolution of epaxial muscle hyperintensity. The absence of abnormal signal changes in the spinal cord and surrounding tissues supports the conclusion that UBE did not induce significant long‐term tissue damage.

## 4 DISCUSSION

This study demonstrated that UBE is a feasible and safe approach for performing MHL in the TL spine in dogs. The first hypothesis was partially accepted. While MHL was technically feasible with both portal positions, Group B showed superior visualization and accessibility compared to Group A. Surgical time did not differ between groups. However, the occurrence of dural tears and nerve root injuries in Group A underscores the importance of optimal portal placement for procedural safety and efficiency. The second hypothesis was accepted, as UBE was found to be safe in live dogs, with stable epidural pressure and no postoperative neurologic complications observed.

The use of separate portals with a triangulation method in the UBE enhanced surgical maneuverability, consistent with the principles of arthroscopy.^18^ Two portals, each 1 cm in size and spaced 2 cm apart, were sufficient to accommodate instruments such as the Kerrison rongeur, RF probe, and arthroscopic shaver. In human studies, 2–3 cm spacing is recommended in UBE to avoid interference between the endoscope and instruments.^35^ Similarly, the 2 cm spacing used in our study was adequate, with no significant interference between instruments and the endoscope during the procedure. Furthermore, in the ex vivo setting, UBE was successfully performed across three contiguous levels (T13–L1, L1–L2, L2–3), suggesting technical feasibility for multilevel decompression. However, further study is warranted to validate these findings in clinical settings.

In human MISS, the Kambin triangle is a well‐established anatomical corridor.^27^, ^28^ Recognized as a safe zone for various endoscopic spinal procedures due to its low risk of involving major vascular and neural structures during the procedure.^27^, ^28^ However, to the best of the author's knowledge, this concept has not been previously applied in dogs. We adapted the Kambin triangle for use in canine UBE. After identifying the foramen, bone drilling was initiated within this adapted Kambin triangle. As the surgeon (SN) was right‐handed, the endoscope was held in the left hand and instruments in the right. Although there was no statistical difference in procedure time between right side and left side, right‐sided procedures posed a technical challenge, as the burr partially obstructed the endoscopic view during drilling. This difficulty was particularly evident since all procedures were performed by a single right‐handed surgeon (SN). Further studies are warranted to evaluate whether switching hand dominance improves ergonomics and visualization.

In the ex vivo study, the MP interfered with instruments and endoscope handling in Group A. Contact between the endoscope and the MP restricted its trajectory, reducing depth perception and procedural efficiency. This limitation may have contributed to the two nerve root injuries and one dural tear observed in Group A. While Group B showed superior visualization and accessibility, this advantage may reflect the study's focus on MHL, for which a more lateral approach is particularly well‐suited. However, for surgeries requiring facet joint removal, such as hemilaminectomy, the Group A portal may offer better access through direct joint visualization and palpation.

While the cadaveric phase was essential for evaluating anatomical feasibility and portal positioning, it could not replicate dynamic intraoperative factors such as bleeding and epidural pressure changes caused by continuous irrigation. As noted by Lockwood and his colleagues, cadaveric models are inherently limited in their ability to assess hemorrhagic conditions.^14^ The in vivo phase enabled real‐time monitoring of epidural pressure, evaluation of intraoperative bleeding control, assessment of potential postoperative neurologic complication, and postoperative muscle recovery through MRI, thus addressing critical safety aspects that cadaveric models cannot provide.

In UBE, continuous endoscopic irrigation enables clear visualization despite minor bleeding from muscle and bones, maintaining a clear surgical field. However, this approach has been associated with fluid‐related complications in humans, including headache, neck pain, seizure, and myelitis.^29^, ^30^, ^31^, ^32^ To mitigate these risks, human studies recommend maintaining irrigation pressure below 30 mmHg and emphasize the importance of adequate outflow.^31^, ^32^ To address this in our study, a semi‐tubular retractor was placed in the instrument portal to facilitate outflow, and the irrigation pressure was maintained below 30 mmHg. As a result, intraoperative epidural pressure remained stable at an average of 2 ± 1 mmHg during the procedure, consistent with a previous study reporting a baseline pressure of 2.1 ± 6.1 mmHg.^34^ Future studies are warranted to evaluate the effect of outflow obstruction caused by instrument manipulation.^31^

In human medicine, open lumbar spine surgery is known to cause injury and atrophy to multifidus muscles, often resulting in chronic back pain and functional disability.^36^, ^37^ Both animal and human studies have shown that prolonged retraction and excessive tissue manipulation during open procedures are strongly associated with muscle damage.^38^, ^39^ In contrast, a human study reports that UBE causes less muscle injury than open surgery.^37^ However, comparable UBE data in dogs are limited. In this study, postoperative MRI was used to evaluate muscle injury following UBE. A transient increase in T2‐weighted signal intensity was observed in the epaxial muscles on postoperative day 0, which progressively resolved by day 28. These findings suggest reversible postoperative inflammation and support the muscle‐sparing nature of this minimally invasive approach.^36^, ^38^

This study had several limitations. First, the UBE technique was applied to clinically normal canine specimens, which may not fully reflect the anatomical and pathologic characteristics of dogs with TL spinal disease. Second, the study focused solely on the MHL procedure, limiting the generalizability of the findings to other spinal surgeries. Third, all procedures were performed by a single surgeon (SN), and the influence of increasing surgical experience throughout the study may have affected the results. Fourth, accessibility was assessed by a single evaluator (SN), introducing the possibility of subjective bias despite the use of predefined criteria. Future studies should involve multiple blind evaluators to assess interobserver reliability. Fifth, epidural pressure was monitored in only three in vivo cases, which may be insufficient to fully characterize pressure variability under different conditions. Future studies with larger sample size and disease‐affected models are warranted to validate these findings. Lastly, all procedures were performed on the left side only, based on the objective of in vivo to evaluate irrigation‐related pressure changes and bleeding control, rather than comparing side‐dependent performance.

## 5 CONCLUSION

This study demonstrated that UBE is a feasible and safe approach for performing MHL in the TL spine of dogs. Evaluation of portal positioning revealed that Group B, which is more laterally positioned portal compared to Group A, provided superior surgical visualization and accessibility. Therefore, when performing MHL in TL spine, caution should be exercised when placing portals close to MP, as this may increase the risk of complications such as dural tears or nerve root injury. In conclusion, UBE presents a feasible minimally invasive option for thoracolumbar mini‐hemilaminectomy in dogs and may serve as a basis for future applications in other spinal procedures.

## AUTHOR CONTRIBUTIONS

Nam S, DVM: Designed the concept of the study, developed methodology and established protocols, performed surgical procedure and postoperative assessments, analyzed and interpreted data statistically. Jeon Y, DVM, PhD: Assisted with surgical procedure and contributed to data collection, and critical review of the work. Kim J, MD: Participated in the study design, analysis and interpretation of the research data, and critical review of the work. Jeong J, DVM, PhD: Participated in the study design, and critical review of the work. Jeong S, DVM, PhD: Participated in the study design, and critical review of the work. Lee Y, DVM, PhD: Participated in the study design, radiologic evaluation of CT and MRI images, and critical review of the work. Lee H, DVM, PhD: Supervised the entire research process, reviewed and approved the final manuscript. All authors are aware of their respective contributions and have confidence in the integrity of all contributions.

## FUNDING INFORMATION

The Korea Institute of Planning and Evaluation for Technology in Food, Agriculture and Forestry, and the Ministry of Agriculture, Food and Rural Affairs (grant/award no.: MAFRA 322090).

## CONFLICT OF INTEREST STATEMENT

The authors declare no conflicts of interest related to this report.

## Supporting information


**Video S1:** Intraoperative endoscopic.

## References

[1] Cudia S , Duval J . Thoracolumbar intervertebral disk disease in large, nonchondrodystrophic dogs: a retrospective study. J Am Anim Hosp Assoc. 1997;33(5):456‐460. 9278123 10.5326/15473317-33-5-456

[2] Macias C , McKee W , May C , Innes J . Thoracolumbar disc disease in large dogs: a study of 99 cases. J Small Anim Pract. 2002;43(10):439‐446. 12400641 10.1111/j.1748-5827.2002.tb00010.x

[3] Penning V , Platt S , Dennis R , Cappello R , Adams V . Association of spinal cord compression seen on magnetic resonance imaging with clinical outcome in 67 dogs with thoracolumbar intervertebral disc extrusion. J Small Anim Pract. 2006;47(11):644‐650. 17076787 10.1111/j.1748-5827.2006.00252.x

[4] Hansen HJ . A pathologic‐ Anatomical Study on Disc Degeneration in dog: With Special Reference to the So‐Called Enchondrosis Intervertebralis. Acta Orthop Scand Suppl. 1952;23(11):1‐117. 10.3109/ort.1952.23.suppl-11.01 14923291

[5] Crawford AH , De Decker S . Clinical presentation and outcome of dogs treated medically or surgically for thoracolumbar intervertebral disc protrusion. Vet Rec. 2017;180(23):569. 28283670 10.1136/vr.103871

[6] Moore SA , Tipold A , Olby NJ , Stein V , Granger N , Consortium CSCI . Current approaches to the management of acute thoracolumbar disc extrusion in dogs. Front Vet Sci. 2020;7:610. 33117847 10.3389/fvets.2020.00610 PMC7521156

[7] Brisson BA . Thoracolumbar decompression: hemilaminectomy and mini‐hemilaminectomy (pediculectomy). In: Shores A , Ab B , eds. Advanced Techniques in Canine and Feline Neurosurgery. Wiley; 2023:59‐69.

[8] Mayer HM . Minimally invasive spine surgery. In: Mayer H , ed. Minimally Invasive Spine Surgery: A Surgical Manual. 2nd ed. Springer; 2006:3‐7.

[9] Guevar J , Zidan N , Durand A , Olby NJ . Minimally invasive spine surgery in dogs: evaluation of the safety and feasibility of a thoracolumbar approach to the spinal cord. Vet Surg. 2020;49(Suppl 1):O76‐O85. 31998976 10.1111/vsu.13385

[10] Carozzo C , Maitre P , Genevois JP , Gabanou PA , Fau D , Viguier E . Endoscope‐assisted thoracolumbar lateral corpectomy. Vet Surg. 2011;40(6):738‐742. 21770985 10.1111/j.1532-950X.2011.00862.x

[11] Hwang YH , Lee HC , Lee JH . Operative techniques and preliminary outcomes following percutaneous endoscopic thoracolumbar pediculectomy in dogs. Vet Surg. 2016;45(S1):O84‐O94. 27711963 10.1111/vsu.12569

[12] Moon H‐S , Hwang Y‐H , Lee H‐C , Lee J‐H . Operative techniques of percutaneous endoscopic mini‐hemilaminectomy using a uniportal approach in dogs. J Vet Med Sci. 2017;79(9):1532‐1539. 28757523 10.1292/jvms.17-0148 PMC5627323

[13] Lee SH , Choi SY , Kwak HH , Woo HM . Minimally invasive percutaneous endoscopic thoracolumbar foraminotomy in large‐breed dogs‐a comparative study. Korean J Vet Serv. 2024;47(2):61‐72.

[14] Lockwood AA , Griffon DJ , Gordon‐Evans W , Matheson JA , Barthélémy N , Schaeffer DJ . Comparison of two minimally invasive approaches to the thoracolumbar spinal canal in dogs. Vet Surg. 2014;43(2):209‐221. 24392636 10.1111/j.1532-950X.2014.12098.x

[15] Kamishina H , Nakano Y , Nozue Y , et al. Microendoscopic mini‐hemilaminectomy and discectomy in acute thoracolumbar disc extrusion dogs: a pilot study. Vet Sci. 2021;8(10):241. 34679071 10.3390/vetsci8100241 PMC8539036

[16] Kang J , Lee S , Kim N , Heo S . Minimally invasive mini‐hemilaminectomy‐corpectomy in cadaveric dogs: evaluation of the accuracy and safety of a three‐dimensionally printed patient‐specific surgical guide. BMC Vet Res. 2022;18(1):271. 35831862 10.1186/s12917-022-03374-6 PMC9277833

[17] Heo DH , Lee DC , Park CK . Comparative analysis of three types of minimally invasive decompressive surgery for lumbar central stenosis: biportal endoscopy, uniportal endoscopy, and microsurgery. Neurosurg Focus. 2019;46(5):E9. 10.3171/2019.2.FOCUS197 31042664

[18] Kim JE , Choi DJ , Park EJJ , et al. Biportal endoscopic spinal surgery for lumbar spinal stenosis. Asian Spine J. 2019;13(2):334‐342. 30959588 10.31616/asj.2018.0210 PMC6454273

[19] Pranata R , Lim MA , Vania R , July J . Biportal endoscopic spinal surgery versus microscopic decompression for lumbar spinal stenosis: a systematic review and meta‐analysis. World Neurosurg. 2020;138:e450‐e458. 32147545 10.1016/j.wneu.2020.02.151

[20] Park DY , Upfill‐Brown A , Curtin N , et al. Clinical outcomes and complications after biportal endoscopic spine surgery: a comprehensive systematic review and meta‐analysis of 3673 cases. Eur Spine J. 2023;32(8):2637‐2646. 37079079 10.1007/s00586-023-07701-9

[21] Van Isseldyk F , Padilla‐Lichtenberger F , Guiroy A , et al. Endoscopic treatment of lumbar degenerative disc disease: a narrative review of full‐endoscopic and unilateral biportal endoscopic spine surgery. World Neurosurg. 2024;188:e93‐e107. 38754549 10.1016/j.wneu.2024.05.047

[22] Choi CM , Chung JT , Lee SJ , Choi DJ . How I do it? Biportal endoscopic spinal surgery (BESS) for treatment of lumbar spinal stenosis. Acta Neurochir. 2016;158(3):459‐463. 26782827 10.1007/s00701-015-2670-7 PMC4752582

[23] Kim JY , Ha JS , Lee CK , et al. Biportal endoscopic posterior thoracic laminectomy for thoracic spondylotic myelopathy caused by ossification of the ligamentum flavum: technical developments and outcomes. Neurospine. 2023;20(1):129‐140. 37016861 10.14245/ns.2346060.030 PMC10080434

[24] Son S‐K , Kim DH , Aygun H . The basics and concepts of unilateral biportal endoscopy. In: Heo DH , Park CW , Son SK , Eum JH , eds. Unilateral biportal endoscopic spine surgery. Singapore Pte Ltd, Springer Nature; 2022:9‐19.

[25] Lin G‐X , Huang P , Kotheeranurak V , et al. A systematic review of unilateral biportal endoscopic spinal surgery: preliminary clinical results and complications. World Neurosurg. 2019;125:425‐432. 30797907 10.1016/j.wneu.2019.02.038

[26] Wang Y , Maimaiti A , Tuoheti A , et al. The method of portal making in lumbar unilateral biportal endoscopic surgery with different operative approaches according to the constant anatomical landmarks of the lumbar spine: a review of the literature. Global Spine J. 2024;14(6):1838‐1861. 38314556 10.1177/21925682241230465 PMC11268301

[27] Kambin P , Savitz MH . Arthroscopic microdiscectomy: an alternative to open disc surgery. Mount Sinai J Med. 2000;67(4):283‐287. 11021778

[28] Kambin P , Sampson S . Posterolateral percutaneous suction‐excision of herniated lumbar intervertebral discs: report of interim results. Clin Orthop Relat Res (1976–2007). 1986;207:37‐43. 3720102

[29] Joh J‐Y , Choi G , Kong B‐J , Park HS , Lee S‐H , Chang SH . Comparative study of neck pain in relation to increase of cervical epidural pressure during percutaneous endoscopic lumbar discectomy. Spine. 2009;34(19):2033‐2038. 19675511 10.1097/BRS.0b013e3181b20250

[30] Choi G , Kang HY , Modi HN , et al. Risk of developing seizure after percutaneous endoscopic lumbar discectomy. J Spinal Disord Tech. 2011;24(2):83‐92. 20625320 10.1097/BSD.0b013e3181ddf124

[31] Kang M‐S , Park H‐J , Hwang J‐H , Kim J‐E , Choi D‐J , Chung H‐J . Safety evaluation of biportal endoscopic lumbar discectomy: assessment of cervical epidural pressure during surgery. Spine. 2020;45(20):E1349‐E1356. 32969993 10.1097/BRS.0000000000003585

[32] Hong Y‐h , Kim S‐K , Hwang J , et al. Water dynamics in unilateral biportal endoscopic spine surgery and its related factors: an in vivo proportional regression and proficiency‐matched study. World Neurosurg. 2021;149:e836‐e843. 33540105 10.1016/j.wneu.2021.01.086

[33] Landis JR , Koch GG . An application of hierarchical kappa‐type statistics in the assessment of majority agreement among multiple observers. Biometrics. 1977;33:363‐374. 884196

[34] Son WG , Jang M , Yoon J , Lee LY , Lee I . The effect of epidural injection speed on epidural pressure and distribution of solution in anesthetized dogs. Vet Anaesth Analg. 2014;41(5):526‐533. 24628876 10.1111/vaa.12147

[35] Hwa Eum J , Hwa Heo D , Son SK , Park CK . Percutaneous biportal endoscopic decompression for lumbar spinal stenosis: a technical note and preliminary clinical results. J Neurosurg Spine. 2016;24(4):602‐607. 26722954 10.3171/2015.7.SPINE15304

[36] Tsutsumimoto T , Shimogata M , Ohta H , Misawa H . Mini‐open versus conventional open posterior lumbar interbody fusion for the treatment of lumbar degenerative spondylolisthesis: comparison of paraspinal muscle damage and slip reduction. Spine. 2009;34(18):1923‐1928. 19652636 10.1097/BRS.0b013e3181a9d28e

[37] Ahn J‐S , Lee H‐J , Park EJ , et al. Multifidus muscle changes after biportal endoscopic spinal surgery: magnetic resonance imaging evaluation. World Neurosurg. 2019;130:e525‐e534. 31254694 10.1016/j.wneu.2019.06.148

[38] Gejo R , Matsui H , Kawaguchi Y , Ishihara H , Tsuji H . Serial changes in trunk muscle performance after posterior lumbar surgery. Spine. 1999;24(10):1023‐1028. 10332796 10.1097/00007632-199905150-00017

[39] Gejo R , Kawaguchi Y , Kondoh T , et al. Magnetic resonance imaging and histologic evidence of postoperative back muscle injury in rats. Spine. 2000;25(8):941‐946. 10767806 10.1097/00007632-200004150-00008

